# Defining and detecting fake news in health and medicine reporting

**DOI:** 10.1177/0141076820907062

**Published:** 2020-08-11

**Authors:** Trevor Treharne, Andrew Papanikitas

**Affiliations:** Nuffield Department of Primary Care Health Sciences, University of Oxford, Oxford OX2 6GG, UK

## Introduction

The term ‘fake news’ has risen in prominence since 2016 as a means to discredit politically inconvenient reporting^1^ and more broadly standing for all information which is ‘inaccurate’.^2^ We suggest that deliberate reporting of lies or misleading interpretation of facts poses a threat to informed public decision-making as well as eroding trust in the media and legitimate authorities. Readers struggle to identify real news stories, and in one study of 203 Stanford University students, a majority of 80% thought a ‘native advert’ was a real news story.^3^ This issue is particularly rife in health and medicine reporting. Of the 20 most-shared articles on Facebook with the word ‘cancer’ in the headline, more than half report claims discredited by doctors and health authorities.^4^ When misleading news claims are made, it can be detrimental to public health^5^ and impacts both healthcare utilisation^6^ and medical non-compliance.^7^ It is therefore important to define exactly what fake news entails and how this can be detected in health and medicine reporting. This paper will provide a fake news definition and outline the specific fake news devices which most prominently feature in health and medicine reporting so that fake news can be detected. The paper makes a distinction between fake news and poor reporting in health and medicine news items, arguing that this is not a simple distinction between true and false. Rather that it is the extent that a news items is misleading and creates a *fake view* of health and medicine that constitutes what is considered fake news.

## Defining fake news

Fake news can be simply defined as ‘false news’^8^ or more specifically as ‘fabricated information that mimics news media content in form but not in organizational process or intent’.^9^ While these definitions comprehend the wider phenomena of fake news, when detecting fake news it is important to distinguish between its two strands: entirely fabricated news items and news items that are sufficiently misleading or inaccurate.

### Entirely fabricated news items

These include news items from sites fraudulently masquerading as respected news outlets, but are distributed under bogus URLs, across social media as doctored screen grabs or in emails as plain text. For example, a fake BBC website using the URL ‘bbc-edition.com’ was used to publish fake news promoting Donald Trump and distributing anti-Islamic propaganda, using the BBC branding to mislead readers into the validity of the content.^10^ This area of fake news also includes satire sites which are not intending to present themselves as real news, but as mock takes on news. This includes the likes of *The Daily Mash* and *NewsBiscuit*.

### News items that are sufficiently misleading or inaccurate

These include news items that come from genuine media outlets, professional journalists and online writers who have failed to fact-check and have exaggerated claims or cherry-picked information. This does not include every news item which features some omissions of information. Instead, it is news items that are sufficiently misleading or inaccurate as they have included excessive spin or vital information omissions. It must be conceded that there will be some measure of grey area in this definition, with some stories treading closely to the fake news line without an obvious reason why it has crossed it. There is also the category of ‘poor reporting’ that features poorly written, researched and presented news items that do not entirely cross the line into fake news. It is important to maintain this distinction as the term fake news currently carries some linguistic weight. If every news item which appears short of technical perfection was considered fake news, the term would be weakened, confusing for the general reader and eventually largely meaningless.

## Detecting fake news in health and medicine reporting

The two strands of fake news can be detected by implementing various checks.

### Entirely fabricated news items

There are four areas of validation which can assist in detecting entirely fabricated news items. First, around finding the ‘original source’, as many entirely fabricated news items are distributed as plain text in emails, as doctored screen grabs on social media, or by fake links, the story should be searched on the media publication’s official site or in the hardcopy publication to verify its authenticity. Second, the ‘scope of coverage’, where readers should check if the news story in question appears on a range of reputable sites. A simple Google search will discover if a story has been widely reported and if reputable sources – such as Reuters and Associated Press – have also reported on the news item. Third, it is important to ‘utilise fact-checking sites’, where sites such as Snopes.com and Factcheck.org list fake news stories currently being distributed. For health and medicine reporting, HealthNewsReview.org has a toolkit for detecting fake news. While still susceptible to biases of their own, sites such as Snopes.com use a team of researchers and are also used in an academic setting to test the truth of claims.^11^ Fourth is a ‘general search of publication title’ to check if the news item is from a satire publication; it is useful to research details of the publication and its history.

### News items that are sufficiently misleading or inaccurate

This list represents five highly prominent areas of misleading or inaccurate claims specifically in health and medicine reporting:

**Contextualisation.** When a news item fails to reasonably place new health and medicine research in the context of previous findings in the same area. It must be made clear if new research contradicts mainstream scientific consensus and leading the news item on the new findings is a misleading reporting approach. This may take the form of a story which states ‘Drug X linked to disease Y’, when drug X is an established evidence-based treatment. If a single study has contracted mainstream scientific consensus on drug X, the news item must make it clear this is the case.
**Causality.** When a news item draws a causal relationship that cannot be proven by the original research. If a news item states ‘Food A reduces disease Y’, but the paper merely found a correlation between food A eaters and lower rates of disease Y, this is unduly misleading. The news item should also not take general or mechanistic findings in a paper and apply it to specific areas for the purposes of the appeal of the story. For example, when the paper ‘Individual differences in bitter taste preferences are associated with antisocial personality traits’^12^ resulted in the news headline ‘If you like gin and tonic, you might be a psychopath.’^13^
**Risk**. Reporting of risk should be expressed in absolute as well as relative terms. For example, the *Daily Mail* story ‘Statins can weaken muscles and joints: Cholesterol drug raises risk of problems by up to 20 per cent’^14^ used relative risk when it is based on a study which found that statins increase the risk of muscle problems from 85% to 87%^15^ in terms of absolute risk. This is an area where a news item can be factually correct in real terms, but considered fake news by the misleading conclusions it draws.
**Extrapolation.** When a news item takes research which has used animal models and test tube findings and creates the appearance that the findings can be applied to humans. As only 1% of drugs tested on animals/cell cultures are appropriate for clinical use in patients,^16^ it is misleading to take such early stage research and offer the appearance that it applies to humans.
**Credibility.** The quality of the evidence must be assessed, including if it has been peer-reviewed and which level of evidence it constitutes (systematic reviews versus observational study, for example). Presenting low-quality research as the latest consensus in the science of an area is misleading. Journalists are not expected to perform a scientific evaluation themselves, but rather make a news judgement based on the quality of the evidence. This is no difference to any other area of news journalism, where the quality of the source, information and findings will be evaluated to decide if a story should be covered and how much prominence it should be given.

## Poor reporting, not fake news

There are further aspects of ‘poor reporting’ in health and medicine news items that do not cross the line into fake news. Good health journalism would include these aspects, but an article should not be considered fake news just because they are not included. This is because we have to make reasonable demands on the media and by overusing the tag of ‘fake news’ it is likely to dilute the impact of that term. Three examples of such ‘poor reporting’ are issues around ‘study limitations’, as many trials will list study limitations, while an informed reader could identify further issues by analysing the results. However, it is not necessarily considered fake news to fail to mention these aspects, providing the limitations do not breech any of the fake news indicators outlined in the previous section. There is also the issue of ‘suitability’, where moving beyond the quality of the research, it is important to understand the suitability of the research methods or trial design. For example, a cohort study might be the best method for certain research questions. Due to the highly technical nature of this aspect, it is not considered fake news to fail to mention it. There is then the issue of ‘bias’, as it is possible that a study has been conducted by researchers in a biased fashion (selection bias, performance bias, detection bias, etc.). While these aspects could impact trial results, it is not considered fake news to not prominently note this in a news item. It should be a greater focus of the researchers themselves and the peer review process before the information is made available to journalists.

## Conclusion

We have attempted to draw boundaries around the forms of fake news and the specific strands of fake news in health and medicine reporting. It is hoped by drawing these boundaries, fake news can be more readily detected. The difficulty lies in drawing the line between fake news and poor reporting. This paper’s approach to that division is not a simple split between true and false. Rather, that the most damaging news approaches in terms of the public’s understanding of health and medicine should be considered fake news due to the fake impression it gives of health and science evidence. Fake news should not become an overused catch-all term for every news item which does not report every fact in detail. It is hoped that the five areas of fake news in health and medicine reporting in this paper can operate as a guide for both journalists and readers alike to increase the quality of the media’s coverage of health and medicine, and the public’s understanding of these areas.

## Declarations

### Guarantor

AP
